# A rare giant gastrointestinal stromal tumor of the stomach traversing the upper abdomen: a case report and literature review

**DOI:** 10.1186/1477-7819-10-66

**Published:** 2012-04-27

**Authors:** Lei Zhou, Chang Liu, Ji-Gang Bai, Ji-Chao Wei, Kai Qu, Feng Tian, Ming-Hui Tai, Rui-Tao Wang, Fan-Di Meng

**Affiliations:** 1 Department of Hepatobiliary Surgery, the First Affiliated Hospital, School of Medicine, Xi’an Jiao tong University, Xi’an, 710061, China

**Keywords:** Diagnosis, Gastrointestinal stromal tumor, GIST, Prognostic factor

## Abstract

We present the case of a 66-year-old woman with a huge gastrointestinal stromal tumor of the stomach that traversed her upper abdomen. The predominant abdominal sign was a huge, palpable mass, but there were no other distinctive findings in her physical examination or her routine blood workup, including biochemical markers. It was difficult to judge the origin of the mass upon imaging. Furthermore, radiological findings revealed that the mass had a complex relationship with many major blood vessels. An exploratory laparotomy revealed a huge tumor protruding from the anterior wall of the stomach fundus, on the lesser curvature of the stomach, measuring approximately 21 × 34 × 11 cm in diameter and weighing 5.5 kg. A complete resection was performed and the tumor was characterized on immunohistochemistry as a gastrointestinal stromal tumor of the stomach. Preoperative diagnosis of gastrointestinal stromal tumors can be difficult, and we hope that the presentation of this rare case and literature review will benefit other diagnosing clinicians having similar problems.

## Background

Gastrointestinal stromal tumors (GISTs) are rare cancers, accounting for 0.1 % to 3.0 % of all gastrointestinal neoplasms [1]. In adults, GISTs frequently occur in the stomach (approximately 60 %) and 30 % of them appear in the small intestine. On very rare occasions, GISTs originate from the mesentery, omentum or retroperitoneum outside the gastrointestinal tract [2,3].

GISTs range from incidental lesions a few millimeters in diameter to large masses dozens of centimeters in size and they have a broad range of presentations. Some are identified clinically because they cause symptoms; but most GISTs are asymptomatic and discovered incidentally [4]. A large number of new studies on histological diagnostic criteria, GIST molecular biology and pathogenesis, imaging strategies and surgical and adjuvant treatment have been published [5,6] and consequently more aspects of GISTs have been revealed and understood. In spite of this, in some cases it is still difficult to diagnose GISTs.

In this report, we describe a rare case of a huge GIST of the stomach that had traversed the upper abdomen. To the best of our knowledge, this is the largest tumor reported in the literature. The tumor was completely resected and our department of pathology characterized it through immunohistochemistry (using, for example, CD117, CD34, Dog-1) to evaluate the direction in which it was tending to differentiate.

### Case presentation

Our hepatobiliary surgery outpatient department accepted a 66-year-old woman, who presented with a year-long history of aggravating abdominal discomfort that was mainly associated with distension and symptoms such as epigastric fullness, eructation, decrease of food intake and early satiety. She did not report any loss in body weight. A physical examination revealed a palpable mass in the upper abdomen, extending from the right hypochondrial region to the left hypochondrial region, with ordinary consistency and a smooth surface. Her liver and spleen were not palpable distance from the costal margin, and were not tender on palpation. All routine blood and biochemical markers were normal. The levels of tumor markers assessed, including carbohydrate antigen 125 and carcinoembryonic antigen, were within the normal ranges.

**Figure 1:**
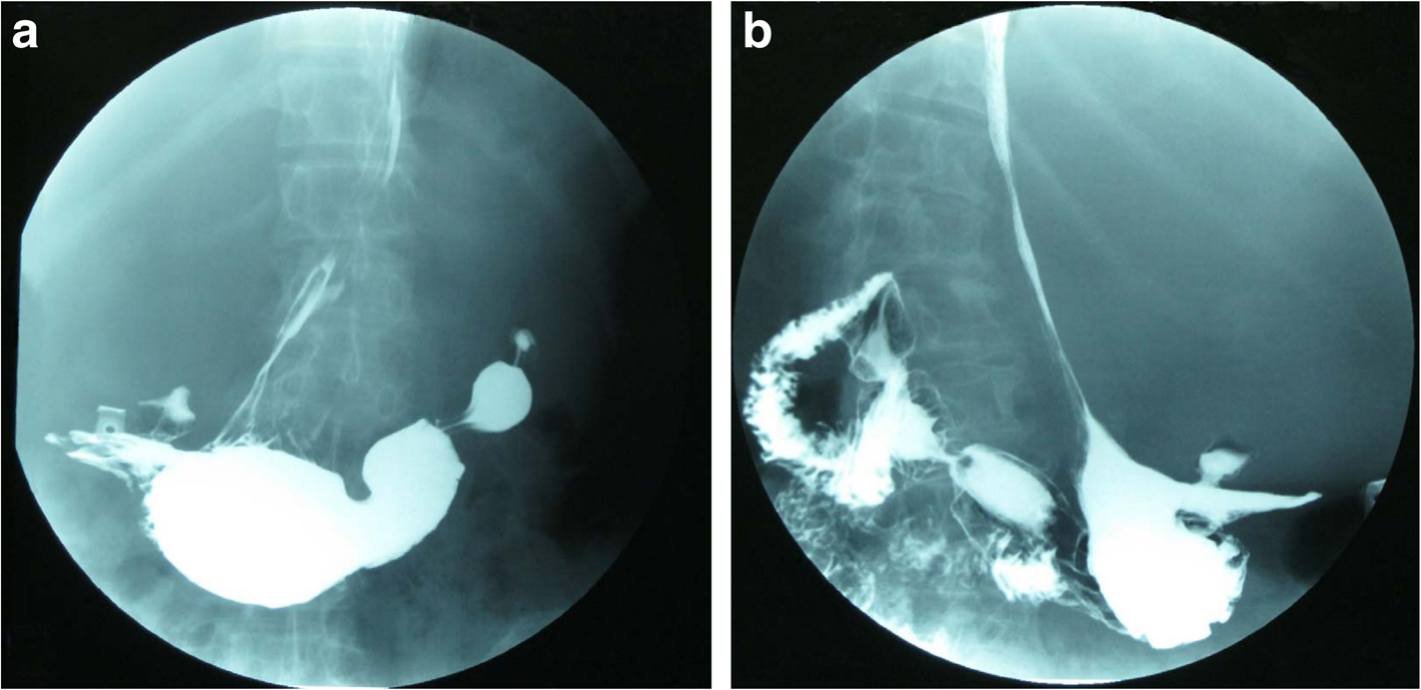
**Upper gastrointestinal barium. (A)** The fundus and body of the stomach are narrowly compressed and a high-density area can be seen in the left-middle abdomen. **(B)** The antrum of the stomach and jejunum are also compressed and infraplacement.

An upper gastrointestinal barium series revealed the antrum, fundus and body of her stomach, as well as the jejunum, were prominently compressed and infraplacement (Figure 1). However, the gastroscope examination did not show any abnormality. An abdominal ultrasound revealed a sharply defined mass with an intact capsule and opaque dark areas of fluid detected in the middle region, suggestive of a huge cystic solid myoma. We were not able to identify any connection between the adjacent organs and the mass because the tumor was too large to obtain a clear view.

**Figure 2:**
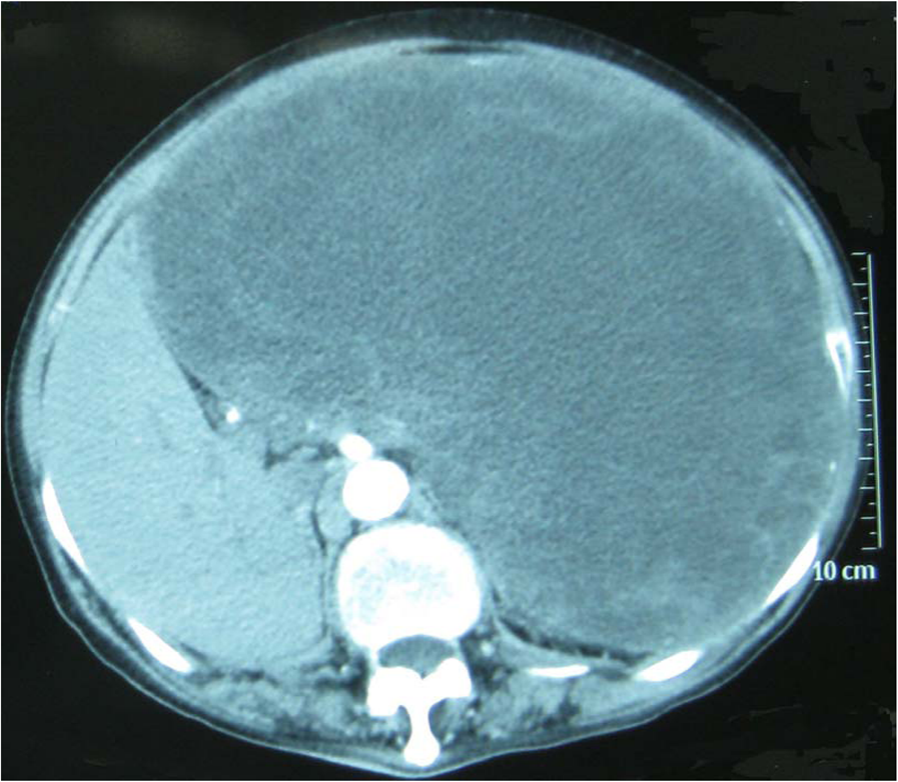
Enhanced abdominal computed tomography shows a large low-density area over the entirety of the left and part of the right liver.

Enhanced abdominal computed tomography (CT) scans demonstrated a deformed liver of abnormal size and structure, and a large low-density region featuring a well-circumscribed border that overlay the entire left liver and partially overlay the right liver. The tumor contained inhomogeneous cystic components mixed with solid elements (Figure 2). In the arterial phase, complete filling of the tumor with contrast material was never observed; patchy enhancement indicated possible tumor necrosis in the low-attenuation areas. The area of lower density also did not enhance completely on delayed scans. There were no signs of lymphadenopathy or liver or pancreatic disease.

**Figure 3:**
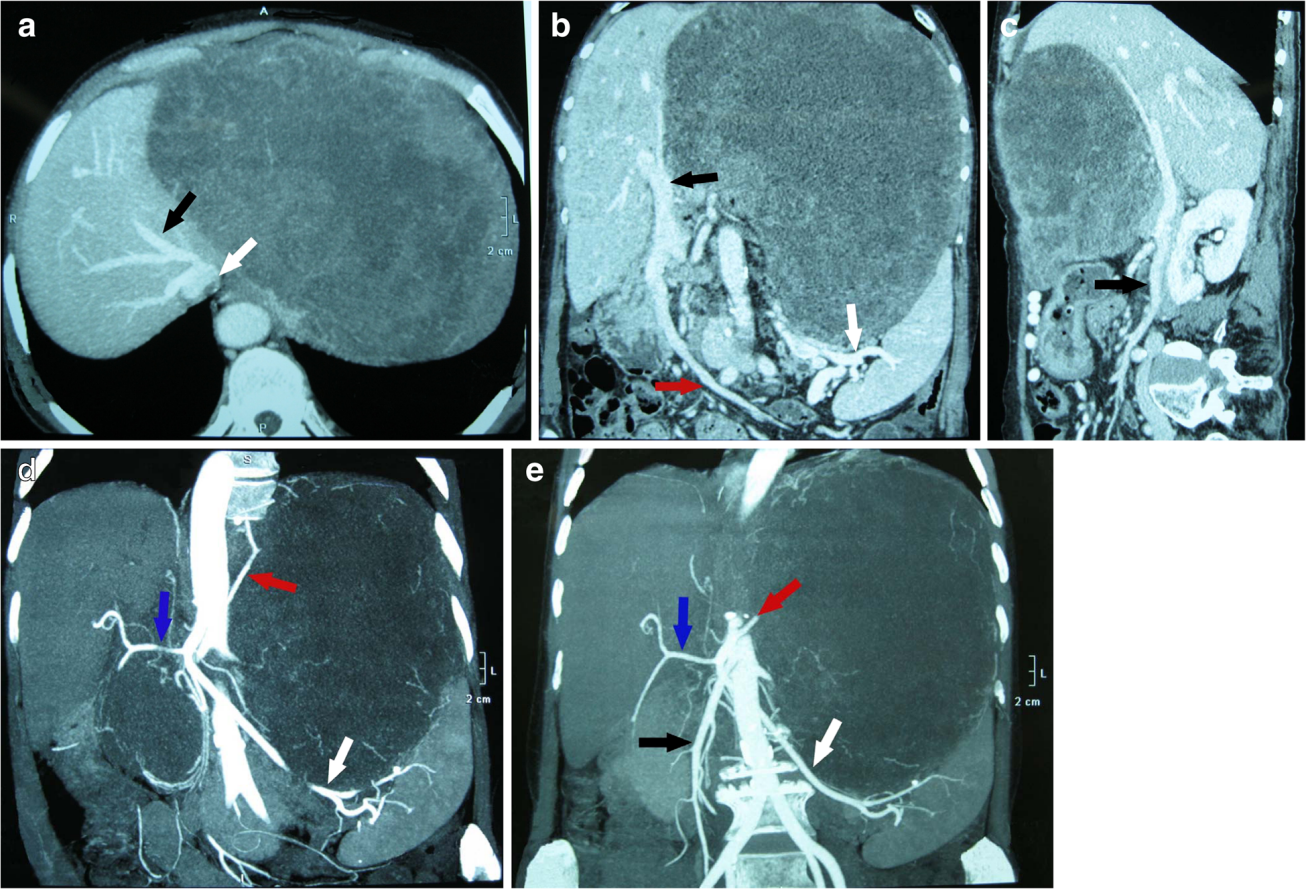
**Computed tomography angiography. (A)** The image illustrates a huge mass measuring about 30 cm in the maximum diameter. The left hepatic vein (black arrow) is compressed to the right by the huge mass. In the second hepatic portal, the inferior vena cava (white arrow) is also compressed. **(B)** The portal vein (black arrow) and superior mesenteric vein (red arrow) are compressed to the right. The trace of the splenic vein (white arrow) is not clear. **(C)** The superior mesenteric vein (black arrow) is compressed. **(D)** The tumor is supplied mainly by branches from the left (red arrow) and right gastric artery, although origin from the common hepatic artery is also possible. The common hepatic artery (blue arrow), left hepatic artery and splenic artery (white arrow) are adjacent to the tumor. **(E)** In addition to the arteries mentioned in (C), the superior mesenteric artery (black arrow) and left renal artery are also next to the tumor and compressed.

CT angiography (CTA) showed that the tumor was supplied mainly by branches from the left and right gastric arteries. The common hepatic artery, left hepatic artery, splenic artery, superior mesenteric artery and left renal artery next to the tumor were compressed. The portal vein, inferior vena cava, left hepatic vein and superior mesenteric vein were also compressed (Figure 3).

**Figure 4:**
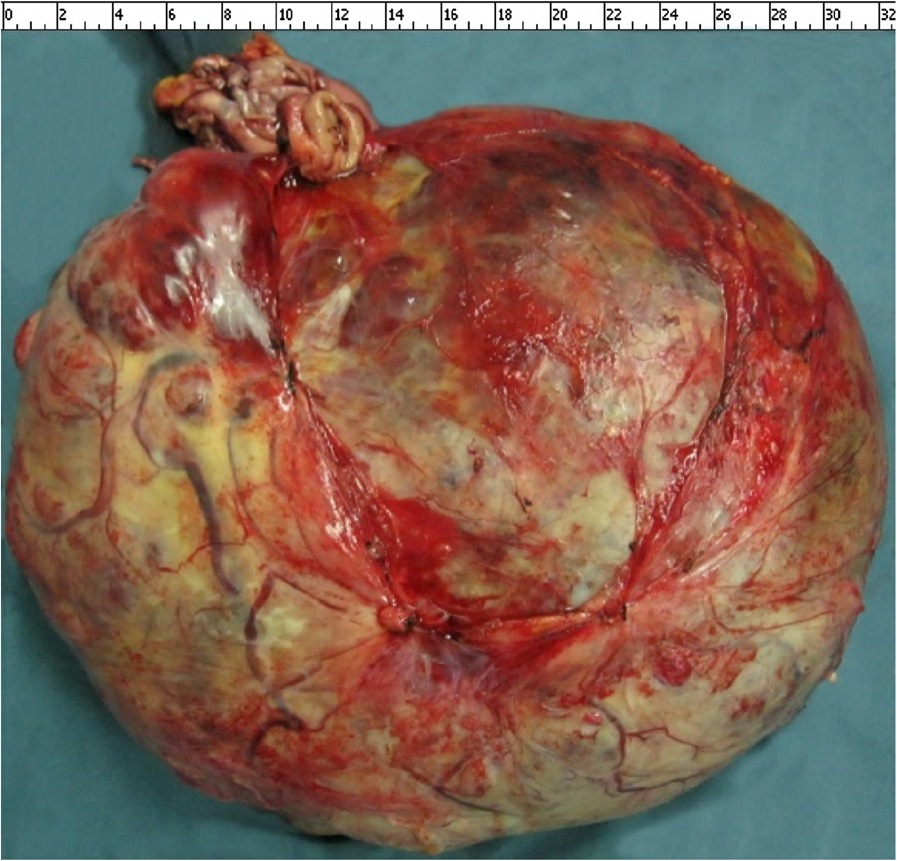
The tumor protrudes from the anterior wall of the fundus, on the lesser curvature of the stomach, measuring 21 × 34 × 11 cm in diameter and weighing 5.5 kg.

An exploratory laparotomy under general anesthesia was performed and revealed a huge, thick-walled tumor that almost filled the abdominal cavity. It appeared to protrude from the anterior wall of the fundus of her stomach, on the lesser curvature. The tumor was well-demarcated from the surrounding organs (liver, spleen, transverse colon), which were displaced but not involved with the tumor. Furthermore, the tumor had no relationship with the adjacent major vessels. In order to completely extirpate the tumor, the proximal stomach and lower esophagus were segmentally resected, and then an esophagogastrostomy was performed. No evidence of liver metastasis, lymphadenopathy or peritoneal metastasis was found (Figure 4). The tumor measured approximately 21 × 34 × 11 cm in diameter and weighed 5.5 kg.

Histopathological examination of the resected specimen revealed a stromal cell neoplasm with necrotic and hemorrhagic areas, as well as a proliferation of spindle cells with a mitotic count of less than five mitoses per 50 high-power fields.

Immunohistochemical analysis revealed the specimen to be CD177 positive, CD34 positive, Dog-1positive, Ki-67 positive (1 %) and S-100 negative. The postoperative course was uneventful and treatment with imatinib mesilate was initiated immediately. Our patient was discharged 20 days after surgery and advised to attend follow-up CT scans of her abdomen in regular three to six months intervals.

## Discussion

GISTs are soft tissue sarcomas. They represent the most common mesenchymal neoplasms of the gastrointestinal tract. Based on an analysis of GIST histology, most of them can be sorted into three major histologic patterns: spindle cell type (70 %), predominantly epithelioid cell type (20 %), or a mixture of both spindle and epithelioid cells [7].

The immunohistochemical features of GISTs are used to confirm diagnosis. Studies have confirmed that approximately 95 % of GISTs are positive for KIT (also known as CD117), which plays a central role in pathogenesis. Gain-of-function mutations in *KIT* lead to constitutive overexpression and autophosphorylation of c-kit, which induces cells toward proliferation and/or away from apoptotic pathways [8]. However, many non-GISTs are also positive for KIT, and about 5 % of GISTs have no detectable KIT expression [9,10].

Many new gene mutations are used in identifying KIT-negative GISTs or to differentiate GISTs from similar tumors. About 80 % of KIT-negative GISTs were found to have a *platelet-derived growth factor receptor alpha* gene (*PDGFRA*) mutation, which results in an epithelioid morphology. This discovery had been used in discriminating between KIT-negative GISTs and other gastrointestinal mesenchymal lesions [11,12]. *BRAF* mutations have been revealed in a small number of high-risk GISTs which develop from intestine lacking *KIT* or *PDGFRA* mutations [13]. A promising calcium-dependent and receptor- activated chloride channel protein, known as DOG1, has emerged as a sensitive and specific marker used in the setting of KIT-negative GISTs [14]. Protein kinase C theta, a downstream effector in the KIT signaling pathway, is used to discriminate between GISTs and leiomyosarcoma or other tumors which have similar histopathology to GISTs [15]. Recently, a novel biomarker called carbonic anhydrase II, which can promote tumor growth by contributing to intracellular alkalization and extracelluar acidification, has been demonstrated to be quite selective to GISTs among mesenchymal tumors [16].

The clinical presentation of GISTs is erratic. About 70 % of patients are symptomatic and GISTs are associated with a broad range of presentations, including early satiety, bloating and some form of gastrointestinal bleeding, either acute or chronic [17]. However, approximately 30 % are asymptomatic or the GIST is detected incidentally at autopsy. There is no physical finding that specifically suggests the presence of a GIST. Although there are several diagnostic modalities available, such as barium examination of the gastrointestinal tract, CT or abdominal ultrasound, none of them can confirm the diagnosis. On some occasions, these examinations prove to be deceptive with regards to tumor identification. As in our case, CT images and an upper gastrointestinal barium series suggested that the tumor possibly originated from the liver or gastrointestinal tract. In addition, the abdominal ultrasound indicated the possibility that the tumor came from the retroperitoneal organs. We could not determine the diagnosis because signs were evident for both possible diagnoses. Under these circumstances, we decided to perform an exploratory laparotomy. In order to explicitly determine the relationship between important vessels, neighboring organs and the mass, CTA examination was performed before the operation.

Because GISTs are usually radioresistant and insensitive to chemotherapeutic agents, surgery remains the main therapy for patients with primary GISTs who have no evidence of metastasis. The tumor in our report was so huge that it almost filled the abdominal cavity, approximately 21 × 34 × 11 cm. Although no such advanced case had been previously reported in the literature, we were able to completely remove the tumor with the proximal stomach and lower esophagus.

There are also many GISTs which have metastases or are unresectable using current technology. Imatinib mesilate, which is an inhibitor of a family of structurally related tyrosine kinase signaling enzymes, is currently the most effective treatment for GISTs [18]. However, some patients are insensitive to imatinib mesilate, as almost immediately after initiation or disease stabilization they then experience disease progression while on medication. In this situation, a multi-kinase inhibitor which inhibits, for example, KIT, PDGFR and vascular endothelial growth factor receptors 1 to 3, such as sunitinib, is indicated for those who fail imatinib treatment [19]. There are a few patients with GISTs who fail both imatinib and sunitinib treatment. In these cases, research has shown that nilotinib, which specifically inhibits KIT, PDGFRA and BCR-ABL, resolves the problem [20].

There is general agreement that tumor size and mitotic count are the most important prognostic factors in GISTs. These two features were the foundation for a consensus approach to risk stratification of GISTs published in 2002 [21]. Subsequently, the criteria for the risk stratification of GISTs have constantly been expanded due to the availability of long-term clinical follow-up. Based on the long-term follow-up of more than 1,600 patients, Miettinen and Lasota [2] suggested guidelines for the risk stratification of primary GISTs based on mitotic index, size and site. Moreover, other pathologic features, including cellularity, mucosal ulceration, and the presence or absence of *KIT* or *PDGFRA* mutations, have been clinically evaluated [22].

## Conclusion

We have described an uncommon huge gastric GIST. According to fundamental surgical principles in the management of gastric GISTs, we completely resected the tumor with the proximal stomach and lower esophagus. The patient has been followed-up after the operation. Furthermore, we will periodically examine the patient and follow support guidelines for medical therapy. We hope the presentation of this rare case could benefit others when they encounter a similar diagnostic problems.

### Consent

Written informed consent was obtained from the patient for publication of this case report and any accompanying images.

## Competing interests

The authors declare that they have no competing interests.

## Authors’ contributions

CL designed the study and LZ drafted the manuscript. CL is the guarantor. All authors contributed to the intellectual context and read and approved the final version.
